# Prevalence of Autoimmune Diseases Among Patients With Psoriasis: A Single Tertiary Center Experience

**DOI:** 10.7759/cureus.60455

**Published:** 2024-05-16

**Authors:** Alanoud R Hakami, Sarah A AlSalman, Narjis J Aljaziri, Bareen Homoud, Mohammad Almohideb

**Affiliations:** 1 Department of Dermatology, King Abdulaziz Medical City, Ministry of National Guard Health Affairs, Riyadh, SAU; 2 Department of Anesthesia, King Abdulaziz Medical City, Ministry of National Guard Health Affairs, Riyadh, SAU; 3 College of Medicine, King Saud Bin Abdulaziz University for Health Sciences, Riyadh, SAU; 4 Department of Dermatology, King Abdullah International Medical Research Center, Riyadh, SAU

**Keywords:** vitiligo, alopecia areata, thyroid, autoimmune, psoriasis

## Abstract

Background

Psoriasis is a common chronic inflammatory skin disease with an autoimmune etiology. Psoriasis has been presumed to be associated with several autoimmune diseases. We sought to determine the prevalence of autoimmune diseases in patients with psoriasis in a large referral tertiary care center.

Methods

This is a retrospective and cross-sectional chart review of patients with confirmed psoriasis diagnoses in the dermatology clinic of King Abdulaziz Medical City, Riyadh, Saudi Arabia. The electronic charts of patients were individually reviewed for autoimmune diseases such as hypothyroidism, hyperthyroidism, alopecia areata, vitiligo, atopic dermatitis, and inflammatory bowel diseases like Crohn’s disease and celiac diseases.

Results

A total of 839 cases were included, 56.4% of whom were females. Most patients were between 31 and 50 years old (37.1%). The most common autoimmune disease was hypothyroidism (6.8%), seen more in females. The second most common autoimmune disease was alopecia areata (3.6%), followed by atopic dermatitis (2.9%). Rheumatoid arthritis, systemic lupus erythematosus, and inflammatory bowel diseases were uncommon in our cohort.

Conclusion

In this single-center retrospective cohort of patients with psoriasis, hypothyroidism and alopecia areata were the most commonly encountered autoimmune diseases. Larger, multi-center studies are needed to evaluate the prevalence of autoimmune diseases among patients with psoriasis.

## Introduction

Psoriasis is a common immune-mediated chronic inflammatory skin disorder that affects both genders equally [1,2]. Psoriasis is considered to be multifactorial, though genetics strongly influences the type and severity of the symptoms [1]. In addition, the immune system plays a significant role in driving the disease process. Patients with psoriasis have an immune modulation in the hyperproliferative keratocytes and infiltrations of dendritic cells, macrophages, neutrophils, and T-cells [3]. Though the pathogenesis of psoriasis is not fully understood, scant evidence suggests that the T-cell immune-mediated process plays a critical role in the disease's development [4]. Previous cohort studies indicated psoriasis patients are more susceptible to developing other autoimmune disorders [5]. The primary autoimmune disorders that have been associated with psoriasis include inflammatory bowel disease (IBD), celiac disease, rheumatoid arthritis, multiple sclerosis, systemic lupus erythematosus (SLE), and autoimmune thyroid diseases [5].

Psoriasis disease has a high tendency to coincide with other autoimmune diseases. This study aims to identify the prevalence of other autoimmune disorders with psoriasis, mainly alopecia areata, SLE, autoimmune thyroid diseases, rheumatoid arthritis, celiac disease, Crohn’s disease, and vitiligo.

## Materials and methods

This study is a retrospective, cross-sectional chart review that was conducted at King Abdulaziz Medical City in Riyadh, Saudi Arabia. To identify patients with psoriasis, a search for psoriasis as the primary diagnosis was conducted in the electronic medical record system. The patients' electronic charts were then individually reviewed to confirm the primary diagnosis of psoriasis. Once the diagnosis was confirmed, each chart was reviewed to check for the presence of autoimmune diseases such as hypothyroidism, hyperthyroidism, alopecia areata, vitiligo, atopic dermatitis, and inflammatory bowel diseases like Crohn’s disease and celiac disease. The study was ethically approved by King Abdullah International Medical Research Center (KAIMRC), Institutional Review Board (IRB) under research protocol RC18/327/R.

Statistical analyses

Numerical data were presented as mean and standard deviation and categorical data were described by counts (n) and percentages (%). Student T-test was used to compare continuous data, and the chi-square test was used to compare categorical data. Statistical analysis was conducted using the statistical software SPSS (IBM Corp., Armonk, NY).

## Results

**Figure 1:**
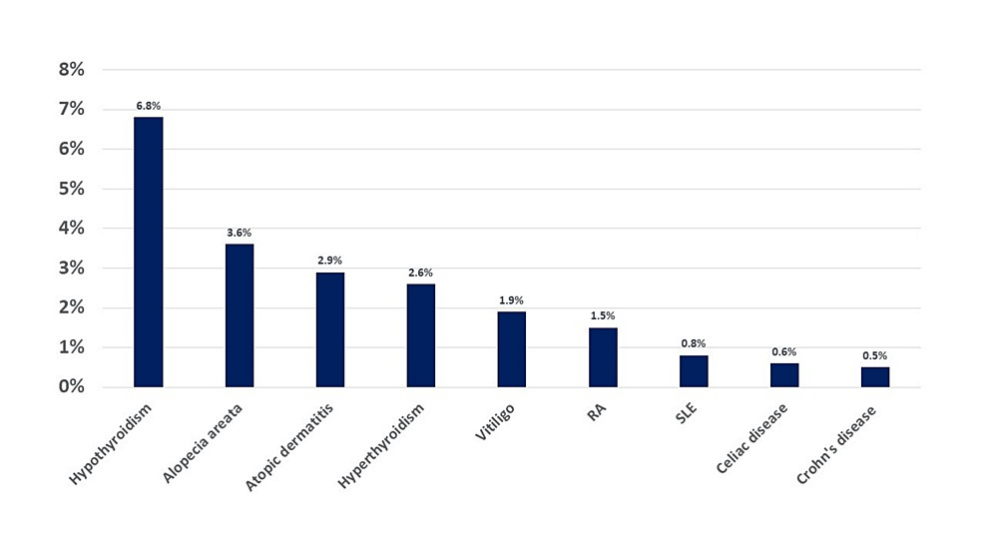
Prevalence of autoimmune diseases among patients with psoriasis RA: rheumatoid arthritis; SLE: systemic lupus erythematosus.

A total of 1098 patients with psoriasis were reviewed, among whom 839 (76.4%) patients had documented autoimmune diseases. Of the cohort, 473 (56.4%) were females. The majority (37.1%) of the patients were between the ages of 31 and 50 years, while 17.6% were children (Table 1). The most associated autoimmune diseases in decreasing orders were hypothyroidism (6.8%), alopecia areata (3.6%), atopic dermatitis (2.9%), and hyperthyroidism (2.6%). Vitiligo, rheumatoid arthritis, SLE, and IBD were seen in less than 2% of the cases (Figure 1). The distribution of autoimmune diseases was similar among males and females except for hypothyroidism, which was more common in females (Table 2).

**Table 1:** Baseline characteristics of the cohort

Characteristics (N = 839)	
Age (years)	Percentage
≤18	17.6% (n=148)
19-30	19.3% (n=162)
31-50	37.1% (n=311)
51+	26% (n=218)
Gender	
Male	43.6% (n=366)
Female	56.4% (n=473)

**Table 2:** Gender difference among autoimmune diseases in patients with psoriasis

	Gender		
Autoimmune disease	Female	Male	P-value
Alopecia areata	3.80%	3.30%	0.684
Systemic lupus erythematosus	0.80%	0.80%	0.967
Hypothyroidism	9.30%	3.60%	0.001
Hyperthyroidism	2.70%	2.50%	0.795
Rheumatoid arthritis	2.10%	0.80%	0.132
Celiac disease	0.60%	0.50%	0.870
Crohn’s disease	0.40%	0.50%	0.797
Atopic dermatitis	3.20%	2.50%	0.539
Vitiligo	2.10%	1.60%	0.618

## Discussion

In this single-center study, autoimmune diseases were common among patients with psoriasis. To elaborate, almost three-quarters of patients with psoriasis were found to have associated autoimmune diseases. The most common autoimmune diseases associated with psoriasis patients were thyroid disorders (9.4%). Particularly, hypothyroidism in 6.8% of the cases, while hyperthyroidism was seen in 2.6%. Autoimmune skin disorders were the second most common findings in patients with psoriasis. The most common skin disorder was alopecia areata (3.6%), atopic dermatitis (2.9%), and vitiligo (1.9%). Connective tissue diseases and IBDs were uncommon in our study.

Multiple observational studies examined the association between psoriasis and autoimmune diseases. Hypothyroidism was reported as high as 18% in patients with psoriasis [3]. Autoimmune skin diseases such as alopecia areata and vitiligo were also described in patients with psoriasis. Patients with psoriasis are twice as likely to have alopecia [6]. Likewise, vitiligo was noticed in 23% of patients with psoriasis [7]. Other studies have also reported the presence of connective tissue diseases such as rheumatoid arthritis, SLE, and IBDs in patients with psoriasis [8-11].

Psoriasis is among the most common immune-mediated diseases genetically linked by sharing common human leukocyte antigen (HLA) alleles [12,13]. In addition, multiple inflammatory pathways play a significant role in the pathogenesis of psoriasis. Dendritic cells and T-cell activation lead to cytokine release, including tumor necrosis factor-alpha, interleukin 23 (IL-23), and interleukin 17 (IL-17) from the neutrophilic granulocytes, keratinocytes, and vascular endothelial cells [14,15]. The same activation has been observed in other autoimmune diseases such as rheumatoid arthritis, thyroiditis, celiac disease, and SLE.

Our study's lower prevalence of autoimmune diseases in psoriasis may be related to our small cohort size or single-center setting. The data were also based on the diagnosis codes documents in the electronic medical records; therefore, some diagnoses may be missing.

Our study has a few limitations. First, it is a single-center retrospective study. Second, the diagnosis of the associated autoimmune diseases is based on the documentation in the medical records. Hence, some of the conditions may be missed or not documented. Finally, there was no comparison between patients with and without associated autoimmune diseases.

## Conclusions

In this single-center retrospective cohort of patients with psoriasis, almost three-quarters had associated autoimmune diseases. Thyroid diseases were the most encountered autoimmune disease (6.8%), followed by skin autoimmune diseases. Connective tissue diseases and inflammatory bowel diseases were uncommon in our cohort. Further multicenter larger studies are needed to understand the extent of autoimmune diseases associated with psoriasis in our region.
